# PD-1/PD-L1 Inhibitors in Cervical Cancer

**DOI:** 10.3389/fphar.2019.00065

**Published:** 2019-02-01

**Authors:** Yuncong Liu, Li Wu, Ruizhan Tong, Feiyue Yang, Limei Yin, Mengqian Li, Liting You, Jianxin Xue, You Lu

**Affiliations:** ^1^West China School of Medicine, Sichuan University, Chengdu, China; ^2^Department of Gynaecological Oncology, Guizhou Provincial People’s Hospital, Guiyang, China; ^3^Department of Thoracic Oncology, Cancer Center, West China Hospital, Sichuan University, Chengdu, China

**Keywords:** cervical cancer, programmed cell death-1/programmed cell death-ligand 1 (PD-1/PD-L1), immune checkpoint inhibitors, immunotherapy, human papillomavirus (HPV)

## Abstract

Cervical cancer is one of the most common gynecological tumors, and the majority of early-stage cervical cancer patients achieve good recovery through surgical treatment and concurrent chemoradiotherapy (CCRT). However, for patients with recurrent, persistent, metastatic cervical cancer, effective treatment is rare, except for bevacizumab combined with chemotherapy. Programmed cell death-1/programmed cell death-ligand 1 (PD-1/PD-L1) inhibitors might be a novel choice to improve the clinical outcomes of these patients. Thus far, some pivotal trials, including Keynote 028, Keynote 158 and Checkmate 358, have indicated established clinical benefit of PD-1/PD-L1 inhibitors in cervical cancer. In light of these data, the FDA has approved pembrolizumab for patients with recurrent or metastatic cervical cancer with disease progression during or after chemotherapy. There are also some ongoing studies that may provide more evidence for the PD-1/PD-L1 pathway as a therapeutic target in cervical cancer. In this review, we have summarized the status and application of PD-1/PD-L1 inhibitors in clinical trials for the treatment of cervical cancer and suggested some future directions in this field.

## Introduction

Cervical cancer is one of the most common gynecological tumors. More than 569,847 women are diagnosed with cervical cancer annually worldwide, resulting in over 311,365 deaths (Bray et al., 2018). Although the incidence of cervical cancer has been greatly reduced by the use of HPV vaccines and cervical cancer screening (Goodman, 2015), cervical cancer is second in terms of morbidity among gynecological tumors in developing countries (Sahasrabuddhe et al., 2012). Over 70% of cervical cancer cases diagnosed in developing countries are locally invasive or metastatic, contributing to the high mortality rate of cervical cancer. The 5-year OS rate of local cervical cancer can achieve approximately 75–85% through effective treatments such as surgery CCRT, etc. (Chen et al., 2015). Nevertheless, the 5-year OS of recurrent, persistent, metastatic cervical cancer is only approximately 15%. The poor prognosis is mainly due to limited therapeutic options (Guitarte et al., 2014). The majority of these patients can only be treated with palliative chemotherapy (Boussios et al., 2016), in which platinum-based chemotherapies were the prior choice (Monk et al., 2009). In 2014, the GOG 240 trial indicated that when bevacizumab was added to the chemotherapy, the ORR was improved from 36 to 48% (Tewari et al., 2014), and the OS could be prolonged from 13 to 17 months for recurrent, persistent, metastatic cervical cancer, thus laying the foundation for the first-line choice of combining bevacizumab with chemotherapy for this population (Tewari et al., 2017). However, for those who progress during the first-line treatment, the lack of effective second-line treatment remains to be the main reason for the high mortality rate (Minion and Tewari, 2018). Currently, immune checkpoint inhibitors (Schumacher and Schreiber, 2015), especially PD-1/PD-L1 inhibitors (Constantinidou et al., 2018), have achieved favorable efficacy in treating multiple solid tumors (Gettinger et al., 2018), including cervical cancer (Borcoman and Le Tourneau, 2017). Accumulating evidence has demonstrated that PD-1/PD-L1 inhibitors may be a promising approach for cervical cancer treatment.

## Immune Checkpoint Inhibitors

**FIGURE 1:**
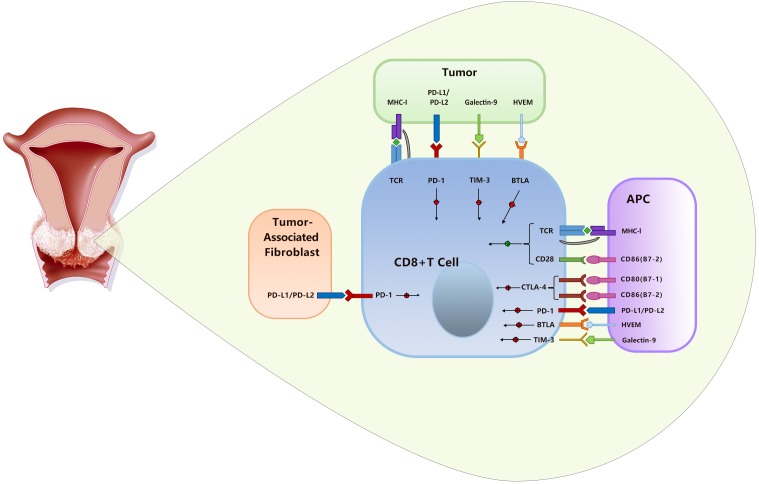
The CTLA-4 and PD-1/PD-L1 pathways in cervical cancer.

Numerous immunomodulatory therapies are being investigated in clinical trials with diverse potential targets, including PD-1/PD-L1, CTLA-4, Tim-3, ICOS, 4-1BB, and OX-40. Among these novel targets, ICOS (Amatore et al., 2018), 4-1BB (Compte et al., 2018), and OX-40 (Polesso et al., 2018) are costimulatory receptors, while PD-1/PD-L1 (Raedler, 2015), CTLA-4 (Lheureux et al., 2018), and Tim-3 (Gorris et al., 2018) are negative immune regulators of T cells. Currently, only CTLA-4 inhibitors (Hodi et al., 2010) and PD-1/PD-L1 inhibitors (Bagcchi, 2014) have been approved by the FDA. CTLA-4 integrates with the costimulatory molecules CD80 (B7-1) and CD86 (B7-2) that express on the surfaces of APCs (Fife and Bluestone, 2008), while PD-L1 is expressed on a wide variety of cell types, including tumor-associated fibroblasts, tumor cells, APCs, etc. (Boussiotis, 2016). As a result, CTLA-4 inhibits T cell activation within secondary lymphoid organs (Kurup et al., 2017), but PD-1/PD-L1 chiefly regulates T cell function within peripheral tissues and the tumor microenvironment (Pardoll, 2012). Therefore, PD-1/PD-L1 signaling is more specific to tumor than CTLA-4 signaling, and inhibitors of PD-1/PD-L1 may cause less damage to healthy tissue (Boussiotis, 2016; Minion and Tewari, 2018) (Figure 1).

Based on the above mechanism, ipilimumab (monoclonal anti-CTLA-4), the first immune checkpoint inhibitor, approved for melanoma, had little clinical benefit until the emergence of pembrolizumab, and the combination of the two drugs further improved treatment efficacy in malignant melanoma (Wang et al., 2017). To date, another mAb for CTLA-4, tremelimumab, has not been approved for the treatment in any type of cancer. However, mAbs targeting PD-1 [pembrolizumab (Paz-Ares et al., 2018), nivolumab (Long et al., 2018), and cemiplimab (Sidaway, 2018)] and PD-L1 [atezolizumab (Hsu et al., 2018), durvalumab (Siu et al., 2018), and avelumab (Le Tourneau et al., 2018)] have presented clinical advantages in malignant melanoma, advanced NSCLC, urothelial cancer (Zhang and Li, 2018) and other tumors (Lim et al., 2018) (Table 1). In addition, extensive research has been carried out on gynecological tumors, such as ovarian cancer (Liu and Zamarin, 2018) and breast cancer (Julia et al., 2018), and clinical researches on cervical cancer are ongoing. At present, some initial results have been achieved.

**Table 1:** The characteristics of the clinical application of monoclonal antibodies (mAbs) of immune checkpoint inhibitors in cervical cancer.

Target	Drug (trade name)	Antibody type	Formerly name	Manufacturer	Time to market (FDA)	Indications
CTLA-4	Ipilumumab (Yervoy)	IgG1	–	BMS	March, 2011	Melanoma, colorectal cancer, renal cell carcinoma
	Tremelimumab	IgG2	Ticilimumb, CP-675,206	Pfizer	–	Undergoing human trials has not attained approval for any
PD-1	Pembrolizumab (Keytruda)	IgG4	MK-3475 Lambrolizumab	MSD	September, 2014	Advanced melanoma, non-small cell lung cancer, Hodgkin’s lymphoma, and head and neck SCC^1^
	Nivolumab (Opdivo)	IgG4	BMS-9365580 NO-4538	BMS	December, 2014	Metastatic melanoma, squamous non-small cell lung cancer, renal cell carcinoma
	Cemiplimab (REGN2810)	IgG4	–	Sanofi	September, 2018 (EMA^2^)	squamous cell skin cancer (EMPOWER-CSCC 1)
PD-L1	Durvalumab (Imfiniz)	IgGlK	–	AstraZeneca	May, 2017	Bladder cancer, NSCLC^3^
	Atezolizumab (Tecentriq)	IgGl	–	Roche	April, 2016	Lung cancer, bladder cancer, advanced triple negative breast cancer


*^1^SCC, squamous cell cancers; ^2^EMA, European Medicines Agency; ^3^NSCLC, non-small cell lung cancer.*

## Theoretical Basis for PD-1/PD-L1 Inhibitors in Cervical Cancer

The PD-1/PD-L1 axis is one of the most well-known immune-checkpoint pathways with a mechanism of immune evasion for cancer cells and thus inhibiting the immune response in various kinds of solid tumors, including cervical cancer Cancer Genome Atlas Research Network et al. (2017). In brief, PD-L1 expresses on the surface of cervical tumor cells, APCs and TILs, while the PD-1-positive cells were mostly identified as T cells in the stroma of cervical tumors. For the expression of PD-1 in the tumor stroma of cervical cancer, Meng et al. (2018) reported that 60.82% (59/97) of the patients exhibited PD-1 expression, while another study showed PD-1 expression in 46.97% (31/66) of the patients (Feng et al., 2018).

To date, numerous studies have investigated the expression of PD-L1 in cervical cancer (Yang et al., 2013; Chen et al., 2016). The expression of PD-L1 has been reported in 34.4–96% of cervical carcinoma tissues, while expression of PD-L1 in histologically normal cervical tissues was rarely found (Enwere et al., 2017). Opal Reddy et al. (2017) showed that PD-L1 expression was positive in 32 of 93 (34.4%) cervical carcinoma samples, subcategorically in 28 of 74 (37.8%) SCCs, 2 of 7 (28.6%) adenosquamous carcinomas, and 2 of 12 (16.7%) endocervical adenocarcinomas. In another study, PD-L1 expression was found in 96% of the samples (Enwere et al., 2017). Specifically, for cervical SCC, PD-L1 expression was found in 80% (56/70) cases (Mezache et al., 2015). In the TCGA database for cervical SCCs, the amplification or gain of PD-L1 was found in 28 of 129 (22%) cases (Dijkstra et al., 2016). In addition, PD-L1 can also be expressed on TILs, which plays a role in antitumor response inhibition. A study found that for cervical SCCs samples, the expression rates of PD-L1 on cancer cells and TILs were 59.1 and 47.0%, respectively (Feng et al., 2018). Collectively, these data suggest that both PD-L1 and PD-1 are widely expressed in cervical cancer tumor cells and stroma, providing potential therapeutic targets for PD-1/PD-L1 inhibitors.

Notoriously, persistent HPV infection is involved in the pathogenesis of cervical cancer and is related to its prognosis. Several teams have interrogated whether HPV infection could affect PD-L1 expression in cervical cancer and found that HPV positivity was positively correlated with increased PD-L1 expression (Mezache et al., 2015; Liu et al., 2017).

Considerable effort has been made to dissect the underlying mechanism of the association between HPV status and PD-L1 expression in HPV-related solid tumors, mainly HNSCC and cervical cancer. In HPV-HNSCCs, membranous expression of PD-L1 and significant increased levels of mRNA of IFN-γ were found in the tonsillar crypts, As tonsillar crypts witnesses the initial HPV infection, and IFN-γ induces PD-L1 expression, this evidence might support the role of the PD-1/PD-L1 interaction in creating an “immune-privileged” site for initial viral infection and subsequent adaptive immune resistance (Franzen et al., 2018). In another study, DNA methylation of PD-L1 was inversely correlated with PD-L1 mRNA expression (*p* ≤ 0.002) and was further significantly associated with HPV infection in the TCGA cohort, indicating that DNA methylation of PD-L1 is associated with transcriptional silencing and HPV infection in HNSCCs (Balermpas et al., 2017). In cervical cancer, Qin et al. (2017) indicated that HPV-induced somatic mutations and a multitude of neoantigens, which played a crucial role in the inhibitory tumor microenvironment and could lead to notable alterations among checkpoint-related genes such as CTLA-4, PD-1, and PD-L1. Specifically, PD-L1 showed a positive correlation with ENO1, PRDM1, OVOL1, and MNT, all of which are related master regulators of HPV16 E6 and E7 (Qin et al., 2017). Of note, a single-arm, phase II study investigated durvalumab in patients with recurrent/metastatic HNSCCs (*n* = 112) and found that HPV-positive patients had a higher response rate and better survival than that of the HPV-negative patients (Zandberg et al., 2018). Nevertheless, for cervical cancer, the association of HPV status and the efficacy of PD-1/PD-L1 inhibitors is not yet certain due to the paucity of available data.

Several studies have probed the role of PD-L1 expression in the prognosis and therapeutic efficacy of cervical cancer. These results separately proved that an increase in PD-L1 expression was positively associated with tumor metastasis (Yang et al., 2017), tumor progression (Hsu et al., 2018) and poor prognosis in cervical cancer (Heeren et al., 2016). In this regard, the negative relationship between HPV infection and the clinical outcomes of cervical cancer may be partially attributed to the PD-L1 expression induced by HPV infection (Yang et al., 2017). For patients with locally advanced cervical adenocarcinoma and adenosquamous carcinoma treated with CRT, the underexpression of PD-L1 was a prognostic factor for tumor relapse (*p* = 0.041), indicating that PD-L1 expression might be a novel biomarker for CRT outcome (Lai et al., 2017).

## Clinical Research Outcomes of PD-1/PD-L1 Inhibitors in Cervical Cancer

Since 2015, multiple clinical trials have been conducted to explore the application of PD-1/PD-L1 antibodies in cervical cancer. To date, four studies have yielded preliminary results (Table 2). Keynote 028 (a phase Ib study) and Keynote 158 (a phase II study) evaluated pembrolizumab at the dose of 10 mg/kg and 200 mg/kg, respectively, in recurrent, metastatic cervical cancer. In Keynote 028 (Frenel et al., 2017), 24 patients were enrolled, and the overall response rate (RECIST v1.1) was 17% (95% CI: 5 to 37%). In terms of toxicity, 5 patients experienced grade 3 AEs (NCI-CTCAE 3.0), while no grade 4 AEs was observed. In Keynote 158 (Schellens et al., 2017), 98 patients with recurrent or metastatic cervical cancer were enrolled. With a median follow-up time of 11.7 months, the ORR in 77 patients was 14.3% (95% CI: 7.4 to 24.1%), including 2.6% of the patients with CRs and 11.7% of patients with PRs, whereas no response was observed in patients without PD-L1 expression in tumor cells. The most frequent serious adverse reactions included anemia (7%), fistula (4.1%), hemorrhage (4.1%), and infection (4.1%). Based on Keynote 158, the FDA approved pembrolizumab on June 12, 2018, for advanced cervical cancer with disease progression during or after chemotherapy^1^. Checkmate 358 (Hollebecque et al., 2017) (phases I–II studies) adopted nivolumab (200 mg/kg q2w) for the treatment of recurrent, metastatic cervical cancer and resulted in an ORR of 26.3%. The disease control rate was 70.8%. The related grades 3–4 toxic effects included hyponatremia, syncope, diarrhea, and hepatocellular injury. From these three studies, pembrolizumab and nivolumab showed promising antitumor effects and were well-tolerated in patients with recurrent or metastatic cervical cancer. However, due to a limited follow-up time, PFS and OS were not reported. Additionally, the REGN2810 study (Papadopoulos et al., 2016), a phase I multicenter study, assessed REGN2810 (a PD-1 mAb) as a monotherapy and in combination with hfRT, in combination with cyclophosphamide (CTX) or with CTX + hfRT in patients with advanced solid tumors, including cervical cancer. This study adopted a dose escalation design, and as of February 2016, no dose-limiting toxicity (DLT) was observed. The most common treatment-related AEs were fatigue (*n* = 14, 24.1%), arthralgia (*n* = 7, 12.1%), and nausea (*n* = 6, 10.3%). Additionally, 4 patients experienced grade ≥ 3 AEs. For 9/22 (40.9%) patients who received REGN2810 + hfRT and 2/21 (9.5%) patients who received REGN2810 monotherapy, they were determined to have partial/uPRs, suggesting that the treatment response was augmented by the addition of hfRT.

**Table 2:** Clinical research outcomes on PD-1/PD-L1 inhibitors in cervical cancer.

Study	Author	Study population (n)	Phase	Treatment arm(s)	Principal results	Toxicity	Significance
REGN2810	Papadopoulos et al., 2016	Advanced solid tumors	I	Cemiplimab	62.8% patients had disease control	No dose-limiting toxicities	Higher response rate when combined with radiation suggesting abscopal responses
Keynote 028	Frenel et al., 2017	Recurrent cervical cancer with PD-L1 positive tumors (24)	Ib	Pembrolizumab 10 mg/kg q2w	ORR^1^ 17% (95% CI: 5–37%)	Grade=3 AE^2^ including rash and proteinuria	Well-tolerated and active in cervical cancer
Keynote 158	Schellens et al., 2017	Recurrent cervical cancer with progression or intolerance to standard therapy (82)	II	Pembrolizumab 200 mg/kg q2w	Preliminary results: ORR^1^ 17% (95% CI: 8–31%); patients with >27 weeks of follow up, ORR 27% (95% CI: 8–55%)	Grades 3–4 AE^2^ included AST/ALT^3^ elevation and pyrexia	Demonstrates activity in cervical cancer and increasing response with a longer duration of follow-up
Checkmate 358	Hollebecque et al., 2017	Recurrent or metastatic HPV^4^-related cancers (19)	I–II	Nivolumab 240 mg q2w	Preliminary results: ORR^1^ 26% (95% CI: 9.1–51.2%) in cervical cancer patients	Grade 3–4 AE^2^ included hyponatremia, syncope, diarrhea and hepatocellular injury	Durable responses demonstrated in cervical cancer patients, with at least 6 months duration


*^1^ORR, objective response rate; ^*2*^AE, adverse event; ^*3*^AST/ALT, aspartate transaminase/alanine transaminase; ^*4*^HPV, human papillomavirus.*

## Ongoing Clinical Research on PD-1/PD-L1 in Cervical Cancer

As of September 2018, 11 clinical trials have been conducted, mainly in patients with persistent, recurrent, or metastatic cervical cancer, with only three studies on patients with locally advanced cervical cancer. Twenty to thirty cases were intended to be included in the majority of these studies, while there were only three studies (Keynote 826, GOG 3016/ENGOT-cx9, and NCT03556839) in which more than 200 cases were intended to be included. Except for the two studies (IMMUVIX, GHR002) aimed at exploring the immune status of PD-1/PD-L1 in patients with locally advanced cervical cancer, the remaining 12 studies all looked into the applicability of PD-1/PD-L1 inhibitors in cervical cancer. Of these 12 studies, there are 2 studies on nivolumab, 2 on pembrolizumab, 4 on durvalumab, 2 on atezolizumab, 1 on cemiplimab (REGN2810) and 1 on AGEN2034. For PD-1 inhibitors, the difference between the 2 studies on nivolumab is the study population. NRG-GYO-02 was conducted in patients with persistent, recurrent, or metastatic cervical cancer, while the NiCOL study enrolled more patients with locally advanced cervical cancer. The main difference between the two studies on pembrolizumab is that KEYNOTE-826 adopted pembrolizumab in combination with chemotherapy versus placebo, while PAPAYA mainly adopted pembrolizumab in combination with platinum and radiotherapy. The GOG 3016/ENGOT-cx9 (EMPOWER-Cervical) study is an important phase III clinical study to advance the clinical application of cemiplimab (REGN2810) in advanced cervical cancer. NCT03104699 is a phase I/II clinical study on AGEN2034, another PD-1 inhibitor, in advanced solid tumors that includes 75 cases of cervical cancer. In terms of treatment combinations, tremelimumab (a fully human mAb against CTLA-4), Vigil vaccine for cervical cancer, bevacizumab, and chemotherapy were paired with PD-1/PD-L1 inhibitors throughout these studies (Table 3).

**Table 3:** Ongoing clinical research on PD-1/PD-L1 in cervical cancer.

Clinical trial code	Study	Study population (n)	Phase	Treatment arm(s)	Primary outcome measures	Secondary outcome measures
NCT02257528	Nivolumab in Treating Patients with Persistent, Recurrent, or Metastatic Cervical Cancer (NRG-GYO-02)	Recurrent or metastatic cervical cancer (25)	II	Nivolumab	ORR^1^ [5 y]; AE^2^ [100 d]	PFS^3^ [5 y], OS^4^ [5 y]
NCT03298893	Nivolumab in Association with Radiotherapy and Cisplatin in Locally Advanced Cervical Cancers Followed by Adjuvant Nivolumab for up to 6 Months (NiCOL)	Locally advanced cervical cancer (21)	III	Nivolumab	DLT^5^ [11 w]	ORR^1^ [2 m], PFS^3^ [2 y], DFS^6^ [2 y], SAE^7^ [100 d], AE^2^ [100 d], etc.
NCT03257267	Study of REGN2810 in Adults with Cervical Cancer (GOG 3016/ENGOT-cx9) (EMPOWER-Cervical)	Recurrent or metastatic platinum-refractory cervical cancer (436)	III	Cemiplimab (REGN2810)	OS^4^ [32 m]	PFS^3^ [32 m], ORR^1^ [32 m], DOR^8^ [32 m], Quality of life (QOL) [100 w]
NCT03104699	Phase 1/2 Study of AGEN2034 in Advanced Tumors and Cervical Cancer	Advanced cervical cancer (75)	I–II	AGEN2034	DLTs^5^ [3 w], MTD^9^ [1 y], BOR^10^ [1 y]	Cmax^11^ [1 y], AUC^12^ [1 y], PFS^3^ [1 y], DOR^8^ [1 y], OS^4^ [1 y]
NCT03518606	Metronomic Oral Vinorelbine Plus Anti-PD-L1/Anti-CTLA4 ImmunothErapy in Patients with Advanced Solid Tumors (MOVIE)	Advanced solid tumors (150) including cervical cancer	I–II	Durvalumab+Tremelimumab+metronomic Vinorelbine	Phase I: MTD^9^ and RP2D^13^ [9 m] Phase II: CBR^14^ [24 m]	None
NCT03556839	Platinum Chemotherapy Plus Paclitaxel with Bevacizumab and Atezolizumab in Metastatic Carcinoma of the Cervix	Carcinoma of the cervix, stage IVB (404)	III	Atezolizumab	OS^4^ [48 m]	PFS^3^ [48 m], ORR^1^ [48 m], DOR^8^ [48 m], AE^2^ [48 m], etc.
NCT01975831	A Phase 1 Study to Evaluate MEDI4736 in Combination with Tremelimumab	Solid tumors (106) including cervical cancer	I	MEDI4736 (Durvalumab)+Trem elimumab	AE^2^ [1 y]	AUC^12^, Cmax^11^ [15 m], PFS^3^ [15 m], OS^4^ [15 m], etc.
NCT02914470	Pilot Study of Durvalumab and Vigil in Advanced Women’s Cancers (PROLOG)	Solid tumors (12) including cervical cancer	I	Durvalumab and Vigil	Toxicity [30 d]	ORR^1^ [120 m]
NCT02725489	Pilot Study of Durvalumab and Vigil in Advanced Women’s Cancers	Solid tumors (15) including cervical cancer	II	Vigil+durvalumab	AEs^2^ [90 d]	ORR^1^ [12 m], Disease status [12 m], IFNγ-ELISPOT conversion rate [12 w]
NCT02921269	Atezolizumab and Bevacizumab in Treating Patients with Recurrent, Persistent, or Metastatic Cervical Cancer	Recurrent, persistent, or metastatic cervical cancer (22)	II	Atezolizumab+Bevac izumab	ORR^1^ [2 y]	PFS^3^ [2 y], OS^4^ [2 y] AE^2^ [30 d], PD-L1, etc.
NCT03635567	Efficacy and Safety Study of First-line Treatment with Pembrolizumab (MK-3475) Plus Chemotherapy Versus Placebo Plus Chemotherapy in Women with Persistent, Recurrent, or Metastati Cervical Cancer (MK-3475-826/KEYNOTE-826)	Cervical cancer (600) c	I–II	Pembrolizumab	PFS^3^ [2y] OS^4^ [2 y]	ORR^1^ [2 y], DOR^8^ [2 y], etc.
NCT03144466	A Study of Pembrolizumab And Platinum with Radiotherapy in Cervix Cancer (PAPAYA)	Cervical cancer (26)	I	Pembrolizum	MTD^9^ [2 y] ab Efficacy [2 y]	OS^4^ [2 y], PFS^3^ [2 y], etc.
NCT03255252	Assessment Study to Evaluate Specific Immune Response in Locally Advanced Cervix Cancer After Radio-chemotherapy (IMMUVIX)	Cervical cancer (100)	II	Cisplatin	Expression of CD8+CD39+PD1+	Effect on 1-year DFS^6^ of other putative biomarkers (CD73, CD39, PD1 and Tim3)
NCT03559803	A Prospective Study of Monitoring Immune Response in Locally Advanced Cervix Cancer(GHR002)	Cervical cancer(50)	Not appli cable	Cisplatin	PD-L1 [3w, 2 m]	PD1+CD4+T [3w, 2 m], PD1+CD8+T [3w, 2 m], TCR[3w, 2 m]


*^1^ORR, objective response rate; ^*2*^AE, adverse event; ^*3*^PFS, progression-free survival; ^*4*^OS, overall survival rate; ^*5*^DLT, dose limiting toxicity; ^*6*^DFS, disease-free survival; ^*7*^SAE, serious adverse event; ^*8*^DOR, duration of response; ^*9*^MTD, maximum tolerated dose; ^*10*^BOR, best overall response; ^*11*^Cmax, maximum plasma concentration; ^*12*^AUC, area under curve; ^*13*^RP2D, phase II recommended dose; ^*14*^CBR, clinical benefit response.*

## Conclusion

Although there are a few studies suggesting the potential feasibility of PD-1/PD-L1 inhibitors for the treatment of cervical cancer, a consideration should be made for the clinical application of PD-1/PD-L1 inhibitors. The inadequate number of cases included and the insufficient follow-up time are the main defects of all the studies, leading to the unavailability of data regarding OS, PFS, AEs, drug resistance and the treatment mechanism as well. These data are very pivotal not only for obtaining a more convincing result, but also for guiding physicians to select the appropriate patients for PD-1/PD-L1 inhibitors.

Currently, most of these studies, including ongoing studies, are mostly limited to recurrent, persistent, metastatic cervical cancer, which accounts for only a minor portion of patients with cervical cancer. There are several future directions that can be given more attention. First, the latest evidence suggests a clinical benefit of PD-1/PD-L1 inhibitors as neoadjuvant therapy in lung cancer (Lommatzsch et al., 2018). For patients with early-stage cervical cancer, studies in a small sample size can be conducted to investigate PD-1/PD-L1 inhibitors with attempted surgical treatment or to prevent post-operative recurrence. Second, for patients with locally advanced cervical cancer who are not sensitive to CCRT or who relapse in the short term after initial treatment, PD-1/PD-L1 inhibitors may be a useful treatment, and we are looking forward to the research targeting this population. Third, for locally advanced cervical cancer patients, whether PD-1/PD-L1 inhibitors can achieve better therapeutic efficacy in tumors with higher PD-L1 expression before CCRT begins will provide a better understanding of the effects of these inhibitors. Finally, since PD-L1 expression is correlated with HPV status, more studies are warranted to provide further insights into the association of HPV status and the efficacy of PD-1/PD-L1 inhibitors in patients with cervical cancer. Combining the level of HPV DNA with the expression of PD-L1 may also provide a novel predictive biomarker of the efficacy of PD-1/PD-L1 inhibitors and the prognosis of patients with cervical cancer.

## Author Contributions

JX and YoL conceived the review. YuL and LYi searched the literature. YuL, LYi, LW, FY, ML, LY, and RT critically appraised the literature and wrote and all authors approved the final version of the manuscript.

## Conflict of Interest Statement

The authors declare that the research was conducted in the absence of any commercial or financial relationships that could be construed as a potential conflict of interest.

## Footnotes

1 https://www.fda.gov/Drugs/InformationOnDrugs/ApprovedDrugs/ucm610572.htm

## Abbreviations

AE: adverse event
APCs: antigen-presenting cells
CCRT: concurrent chemoradiotherapy
CRs: complete responses
CRT: chemoradiotherapy
CTLA-4: cytotoxic T-lymphocyte-associated protein-4
hfRT: hyperfraction radiotherapy
HNSCC: head and neck squamous cell carcinoma
HPV: human papillomavirus
mAb: monoclonal antibody
NSCLC: non-small cell lung cancer
ORR: objective response rate
OS: overall survival rate
PD-1/PD-L1: programmed cell death-1/programmed cell death-ligand 1
PFS: progression-free survival
PRs: partial responses
SCCs: squamous cell cancers
TILs: tumor infiltrating lymphocytes
uPRs: unconfirmed partial responses
